# Supplementary material for: Mediator subunit MDT-15 promotes expression of propionic acid breakdown genes to prevent embryonic lethality in Caenorhabditis elegans
Source: G3 (Bethesda). 2023 Apr 19;13(6):jkad087. doi: 10.1093/g3journal/jkad087 (PMC10234398; doi:10.1093/g3journal/jkad087)
Supplement: jkad087_Supplementary_Data [file jkad087_supplementary_data.zip › Supplemental_Material_Legends_G3-2022-403999.docx]

**Supplementary Figure legends.**

**Supplementary Figure S1. Transcriptomes from specific gene inactivations. (A)** The scatter plot shows the correlation of differentially expressed genes (Adj.P or PValue<0.05) in *mdt-15(tm2182)* mutant worms and *mdt-15(RNAi)* worms. X and Y axes represent logFC. Pearson r=0.66; p=0. **(B-E)** The volcano plots show the expression of all detected genes in **(B)** *mdt-15(RNAi) vs.* EV, **(C)** *mdt-15(tm2182)* *vs.* WT, **(D)** *nhr-10(tm4695) vs.* WT, and **(E)** *nhr-68(gk708)* *vs.* WT worms. The shunt genes and *mdt-15* are highlighted. X-axis, logFC; Y-axis, -log_10_(PValue or Adj.P, as indicated). Black, PValue or Adj.P <0.05; grey, PValue or Adj.P ≥0.05; blue, highlighted and significantly downregulated; purple, highlighted but not significant.

**Supplementary Tables.**

**Supplementary Table S1.** The table shows the number of unique significantly regulated DEGs from each transcriptome dataset. Significance is defined as PValue or Adj.P <0.05. Up- and down-regulation are defined as logFC>0 and logFC<0, respectively. Study type, relevant GEO accession numbers, and source publications are also listed.

**Supplementary Table S2.** List of unique significantly regulated DEGs (Adj.P<0.05) in *mdt-15(tm2182)* mutants *vs.* WT worms.

**Supplementary Table S3.** List of unique significantly regulated DEGs (PValue<0.05) in WT worms fed *mdt-15(RNAi)* *vs.*EV RNAi control.

**Supplementary Table S4.** List of unique significantly regulated DEGs (PValue<0.05) in WT worms fed *C. aquatica* DA1877 *vs.*HT115.

**Supplementary Table S5.** List of unique significantly regulated DEGs (PValue<0.05) in WT worms fed OP50 supplemented with B12 *vs.* OP50 control.

**Supplementary Table S6.** List of unique significantly regulated DEGs (PValue<0.05) in *nhr-10(tm4695)* mutants *vs.* WT worms.

**Supplementary Table S7.** List of unique significantly regulated DEGs (PValue<0.05) in *nhr-68(gk708)* mutants *vs.* WT worms.

**Supplementary Table S8**. Lists of significantly downregulated genes (Adj.P<0.05 or PValue<0.05, and logFC<0) in the following overlaps: *mdt-15(tm2182)* and *nhr-10(tm4695)* (sheet “mdt-15&nhr-10 p0.05 down”); *mdt-15(tm2182)* and *nhr-68(gk708)* (sheet “mdt-15&nhr-68 p0.05 down”); and the triple overlap between *mdt-15(tm2182)*, *nhr-10(tm4695)*, and *nhr-68(gk708)* (sheet “mdt-15&nhr-10&nhr-68 p0.05 down”).

**Supplementary Table S9**. Lists of overrepresented gene sets (pval<0.005, padj<0.25) obtained by performing ORA on the overlap of significantly downregulated genes (Adj.P<0.05 or PValue<0.05, and logFC<0) in the following overlaps: *mdt-15(tm2182)* and *nhr-10(tm4695)* mutants (sheet “mdt-15&nhr-10 ORA p0.005”); *mdt-15(tm2182)* and *nhr-68(gk708)* (sheet “mdt-15&nhr-68 ORA p0.005”); and the triple overlap between *mdt-15(tm2182)*, *nhr-10(tm4695)*, and *nhr-68(gk708)* (“mdt-15&nhr-10&nhr-68 ORA p0.005”).
