# Supplementary figures and images for: Mediator subunit MDT-15 promotes expression of propionic acid breakdown genes to prevent embryonic lethality in Caenorhabditis elegans
Source: G3 (Bethesda). 2023 Apr 19;13(6):jkad087. doi: 10.1093/g3journal/jkad087 (PMC10234398; doi:10.1093/g3journal/jkad087)

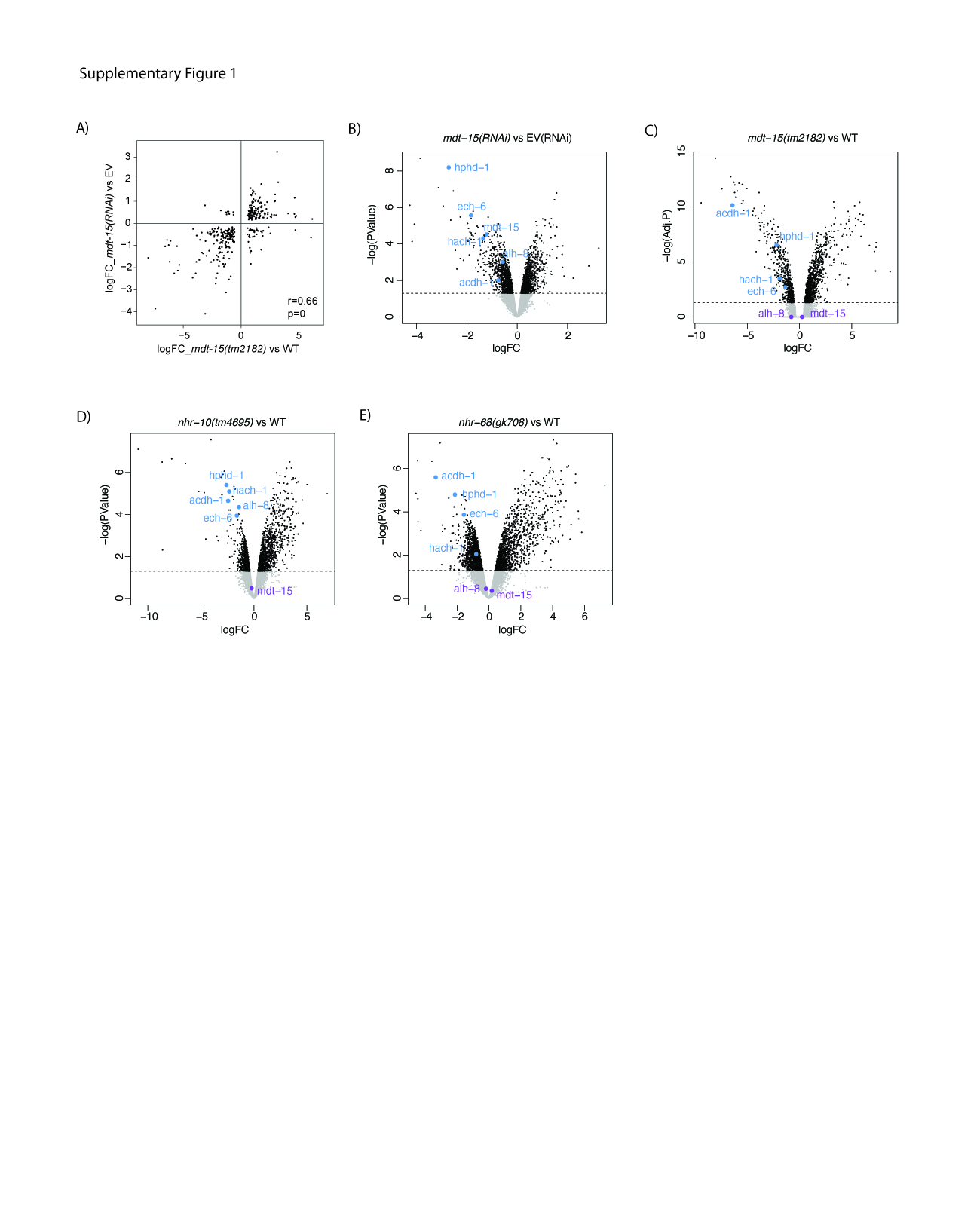

Supplement: jkad087_Supplementary_Data [file jkad087_supplementary_data.zip › Supplemental_Figure_S1_G3-2022-403999.tif]
